# Risk factors for HIV infection among circumcised men in Uganda: a case-control study

**DOI:** 10.7448/IAS.18.1.19312

**Published:** 2015-01-02

**Authors:** Michael Ediau, Joseph KB Matovu, Raymond Byaruhanga, Nazarius M Tumwesigye, Rhoda K Wanyenze

**Affiliations:** 1 Makerere University School of Public Health–CDC Fellowship Program, Kampala, Uganda; 2 Department of Community Health and Behavioural Sciences, Makerere University School of Public Health, Kampala, Uganda; 3 AIDS Information Center, Kampala, Uganda; 4 Department of Epidemiology and Biostatistics, Makerere University School of Public Health, Kampala, Uganda; 5 Department of Disease Control and Environmental Health, Makerere University School of Public Health, Kampala, Uganda

**Keywords:** HIV risk behaviours, male circumcision, risk factors for HIV infection, Uganda, case-control study

## Abstract

**Introduction:**

Male circumcision (MC) reduces the risk of HIV infection. However, the risk reduction effect of MC can be modified by type of circumcision (medical, traditional and religious) and sexual risk behaviours post-circumcision. Understanding the risk behaviours associated with HIV infection among circumcised men (regardless of form of circumcision) is critical to the design of comprehensive risk reduction interventions. This study assessed risk factors for HIV infection among men circumcised through various circumcision approaches.

**Methods:**

This was a case-control study which enrolled 155 cases (HIV-infected) and 155 controls (HIV-uninfected), all of whom were men aged 18–35 years presenting at the AIDS Information Center for HIV testing and care. The outcome variable was HIV sero-status. Using SPSS version 17, multivariable logistic regression was performed to identify factors independently associated with HIV infection.

**Results:**

Overall, 83.9% among cases and 56.8% among controls were traditionally circumcised; 7.7% of cases and 21.3% of controls were religiously circumcised while 8.4% of cases and 21.9% of controls were medically circumcised. A higher proportion of cases than controls reported resuming sexual intercourse before complete wound healing (36.9% vs. 14.1%; *p*<0.01). Risk factors for HIV infection prior to circumcision were:being in a polygamous marriage (AOR: 6.6, CI: 2.3–18.8) and belonging to the Bagisu ethnic group (AOR: 6.1, CI: 2.6–14.0). After circumcision, HIV infection was associated with: being circumcised at >18 years (AOR: 5.0, CI: 2.4–10.2); resuming sexual intercourse before wound healing (AOR: 3.4, CI: 1.6–7.3); inconsistent use of condoms (AOR: 2.7, CI: 1.5–5.1); and having sexual intercourse under the influence of peers (AOR: 2.9, CI: 1.5–5.5). Men who had religious circumcision were less likely to have HIV infection (AOR: 0.4, 95% CI: 0.2–0.9) than the traditionally circumcised but there was no statistically significant difference between those who were traditionally circumcised and those who were medically circumcised (AOR: 0.40, 95% CI: 0.1–1.1).

**Conclusions:**

Being circumcised at adulthood, resumption of sexual intercourse before wound healing, inconsistent condom use and having sex under the influence of peers were significant risk factors for HIV infection. Risk reduction messages should address these risk factors, especially among traditionally circumcised men.

## Introduction

Male circumcision (MC) is the surgical removal of the foreskin of the penis [1] and is practiced around the world for medical, religious and cultural reasons [2]. Circumcision conducted in medical settings with full adherence to medical ethics is termed medical MC. Medical MC, also commonly referred to as safe male circumcision (SMC), is provided confidentially, without coercion and with HIV risk reduction counselling [3]. However, traditional or religious circumcision involving procedures which generally take place outside the formal medical settings [3–5] is performed by providers who are not normally health professionals but may have special training [3]. Traditional and religious circumcision is more often decided by the parents of the boy to be circumcised based on the existing cultural and religious norms [6]. Unlike SMC, traditional circumcision is usually associated with religious or cultural ceremonies [6, 7].

The effectiveness of SMC in reducing the risk of HIV acquisition in heterosexual men has been demonstrated through randomized controlled trials in Uganda, Kenya and South Africa [8–10]. Evidence from mathematical models suggests that full coverage of SMC could avert nearly 6 million new HIV infections and save 3 million lives in sub-Saharan Africa in the next 20 years [2]. Further evidence accumulated over more than 20 years shows that SMC is one of, if not, the most efficacious and cost-effective HIV prevention strategy [11]. Based on this evidence, the World Health Organization (WHO) and United Nations Programme on AIDS (UNAIDS) recommended in 2007 that SMC be considered as part of a comprehensive HIV prevention package [12]. In 2010, Uganda launched a policy to guide SMC implementation and roll-out and has adopted SMC as part of the national HIV prevention strategy [13–15].

In several African societies and certain ethnic groups in other regions, MC is largely carried out for cultural reasons, as an initiation ritual and a rite of passage into manhood [16]. In Bugisu sub-region in Uganda, where this study was conducted, traditional MC is practiced with up to 81% of the adult male population circumcised compared to the national average of 26.7% [17]. HIV prevalence in this region is 4.1% compared to the national average of 7.3%, potentially indicating the protective effect of MC [17]. However, certain cultural practices such as using one knife to circumcise several boys, sexual intercourse shortly after circumcision and before complete wound-healing can increase the risk of HIV transmission [16]. In addition, prolonged wound healing, attributable to traditional ways of cutting the foreskin or complications after traditional MC, has implications for HIV transmission [16]. Two aspects of traditional surgical methods are of concern. First, the amount of foreskin removed through traditional circumcision methods prolongs wound-healing. Secondly, traditional MC is not a standardized procedure, and the amount of foreskin removed varies widely. This has implications for HIV prevention, as only the complete removal of the foreskin is likely to provide partial protection [16]. Techniques used traditionally that result in incomplete or limited removal of the foreskin have been reported in Kenya [18] and Lesotho [19].

Religion is known to be the primary determinant of MC in some settings; for example, almost all Muslims and Jewish males are circumcised [6, 7]. In sub-Saharan Africa, MC for religious and cultural reasons is a relatively common practice, where 28 of 45 countries have a MC prevalence exceeding 80% [20]. Whereas Muslims continue to practice this time-honoured tradition, MC is not nearly as prominent within the Christian populations of Africa [2]. However, the Coptic Christians in Egypt and the Ethiopian Orthodox Christians have retained MC; 97% of Orthodox Christian men in Ethiopia are circumcised [21, 22]. In seven countries in sub-Saharan Africa, where Muslims were among the top five largest religious groups, the circumcision rate among Muslims in each country was greater than the national circumcision rate, on average by a factor of three [23]. This trend is similar in Uganda with up to 98% of Muslims being circumcised compared to the national circumcision prevalence of 26.7% [17]. Some studies in Uganda show that Muslims may have a lower risk profile than other circumcised men [24].

Literature on sexual risk behaviours among circumcised males is mixed. Whereas some studies suggest that SMC is not associated with increased sexual risk behaviours [25], others show that it might engender a sense of being “protected” from HIV infection, thereby resulting in increased risk-taking [26]. However, while medical circumcision integrates HIV counselling and testing and risk reduction counselling which might contribute to its protective effect against HIV infection, traditional forms of circumcision do not include these aspects, and cannot address the risk compensation that may be associated with circumcision. Understanding the risks associated with all forms of circumcision is critical to designing comprehensive messages and other interventions that address traditional, religious and medical MC. In addition, most studies have largely focused on risk factors following medical circumcision. Using a case-control study design, this study explored risk factors associated with HIV infection among men circumcised through various circumcision approaches in Mbale and surrounding districts in eastern Uganda.

## Methods

### Study area

The study was conducted in Mbale and surrounding districts in Bugisu sub-region, in eastern Uganda. The Bugisu sub-region was selected because it is one of the regions where traditional MC is practiced [27]. Mbale also has a significant Muslim population and provides an opportunity to enrol men who were circumcised for religious reasons. The study was conducted in collaboration with the AIDS Information Center (AIC), a local non-government organization that offers, among others, HIV counselling and testing, sexually transmitted infections treatment, antiretroviral treatment, SMC, and sexual and reproductive health services. Respondents were recruited at the facility (AIC Mbale branch) and from the AIC community outreaches within the Bugisu sub-region. Data were collected between April and May 2012.

### Study population

The study population comprised all circumcised adult males (regardless of form of circumcision) aged 18–35 years presenting for HIV services at the AIC branch in Mbale or at any of its community outreaches. The upper age limit of 35 years was considered to reduce the recall period because in the region where this study was conducted, traditional MC is predominant [27] and most of the males are circumcised at the age of 13–15.

### Study design and sample size determination

This was an unmatched case-control study of circumcised males (circumcised at least six months before enrolment into the study). The cases were circumcised HIV-positive men aged 18–35 who came for drug refills and other HIV and AIDS services at AIC Mbale while controls were circumcised men of the same age group who tested HIV negative at the centre and its outreaches. We used the Schlesselman formula [28] in computing the sample size. In the absence of relevant literature on the prevalence of risky sexual behaviours (specifically unprotected sex) among circumcised HIV-positive men in similar study settings, we assumed a prevalence of unprotected sex of 50%. At a prevalence (*p*) of 50%, the Schlesselman formula gives the largest sample size possible because the variance, *p*(1−*p*), is at its maximum. Such a sample size reduces the chances of type II error and thus improves the power of the study.

### Sampling procedure, data collection methods and tools

All males presenting for HIV services either at the AIC main facility in Mbale town or at an AIC community outreach were screened for eligibility. Screening was done by asking the respondents if they were circumcised (including how long ago they were circumcised). All men who reported that they were circumcised at least six months prior to the study and knew their HIV sero-status were enrolled into the study. Eligible HIV-positive circumcised men were enrolled when they came for HIV care and treatment at the AIC facility in Mbale while eligible HIV-negative circumcised men were enrolled after receiving HIV counselling and testing services both at AIC facility and community outreaches. After learning about their HIV status, HIV-negative men were approached and informed about the study, screened for eligibility and invited to participate in the study.

Data were collected using face-to-face interviews by trained interviewers. A pre-tested, semi-structured questionnaire was used to collect data from respondents. The questionnaire had a section pertaining to behaviours in the period before and after circumcision. This distinction was clearly explained to the respondents. The interviews were administered in English and *Lumasaba*, the local language, for most participants who were not fluent in English.

### Study variables

Data were collected on cultural and socio-demographic characteristics (sex, current age and age at the time of circumcision), highest education level attained, ethnicity/tribe, marital status, religion, age at circumcision, form of circumcision (medical, cultural and religious), sexual and other practices before and after circumcision.

### Data management and analysis

All data were entered into a computer using EPIDATA version 3. Data cleaning, coding and analysis were performed using SPSS version 17. Frequencies and proportions were generated to describe the characteristics of the study population. Risk factors for HIV infection were compared between cases and controls using bivariable analysis. At bivariable and multivariable analyses, all variables with *p*<0.05 were considered statistically significant. Unadjusted and adjusted odds ratios (OR) at 95% confidence interval (CI) were used to measure associations. All variables that were significantly associated with HIV infection at bivariable analysis were included into the multivariable logistic regression model. A backward elimination method was then used with an exclusion criteria of *p*>0.05.

### Ethical clearance

The study was approved by the Makerere University School of Public Health Higher Degrees Research and Ethics Committee and the Uganda National Council of Science and Technology.

## Results

A total of 310 circumcised men were enrolled into the study, half of whom were HIV-positive (155 cases) while the other half were HIV-negative (155 controls). Table 1 shows the socio-demographic and behavioural characteristics of the cases and controls.

**Table 1:** Socio-demographic and behavioural characteristics of cases and controls

Socio-demographic characteristics	Cases (HIV+), *n*=155	Controls (HIV−), *n*=155	*p*
Age group			0.01
18–24	35 (22.6%)	58 (37.4%)	
25–29	46 (29.7%)	47 (30.3%)	
30–35	74 (47.7%)	50 (32.3%)	
Marital status			0.01
Previously married	14 (9.0%)	5 (3.2%)	
Never married	61 (39.4%)	85 (54.8%)	
Currently married	80 (51.6%)	65 (41.9%)	
Type of marriage (for currently married)	*N*=80	*N*=65	0.34
Monogamous	47 (58.8%)	58 (89.2%)	
Polygamous	33 (41.2%)	7 (10.8%)	
Religion			0.24
Muslims	33 (21.3%)	25 (16.1%)	
Christians	122 (78.7%)	130 (83.9%)	
Highest education level attained			0.02
Primary and below	57 (36.8%)	38 (24.5%)	
Secondary education and beyond	98 (63.2%)	117 (75.5%)	
Occupation			<0.01
Non-salaried worker	112 (78.7%)	38 (81.3%)	
Salaried/wage worker	33 (21.3%)	37 (18.7%)	
Tribe			<0.01
Non-Bagisu	34 (21.9%)	70 (45.2%)	
Bagisu	121 (78.1%)	85 (54.8%)	
**Behavioural Characteristics**			
Age at circumcision			<0.01
≤17 years	25 (16.1%)	70 (45.2%)	
≥18 years	130 (83.9%)	85 (54.8%)	
Form of circumcision			<0.01
Traditional/cultural	130 (83.9%)	88 (56.8%)	
Religious	12 (7.7%)	33 (21.3%)	
Medical male circumcision	13 (8.4%)	34 (21.9%)	
One knife used to circumcise more than one boy			0.02
No	52 (43.7%)	65 (59.1%)	
Yes	67 (56.3%)	45 (40.9%)	

Compared to controls, cases were older (e.g. 47.7% of cases compared to 32.3% of controls were aged 30–35 years, *p*<0.01), currently married (51.6% vs. 41.9%, *p*<0.01), Bagisu by tribe (78.1% vs. 54.8%, *p*<0.01) and circumcised at 18 years or older (83.9% vs. 54.8%, *p*<0.01). In addition, a higher proportion of cases than controls received traditional circumcision (83.9% vs. 56.8%, *p*<0.01) and reported that only one knife was used to conduct the surgeries at the same circumcision event (56.3% vs. 40.9%, *p*=0.02). However, controls were generally better educated than cases (e.g. 75.5% vs. 63.2%, *p*=0.02) (Table 1).

As expected in this region, traditional MC was prevalent in both the cases and the controls, although it was higher in the cases than controls. The majority of the cases (83.9%) were traditionally circumcised, 8.4% were medically circumcised while 7.7% were religiously circumcised. Among controls, the majority were also traditionally circumcised (56.8%), 21.9% were medically circumcised, while 21.3% were religiously circumcised.

### Descriptive comparison of prevalence of risky sexual practices before and after circumcision among cases and controls

There was no significant difference in the proportion of cases and controls reporting sexual risk behaviours prior to circumcision. On the other hand, there were higher and significant differences in the proportions of cases compared to controls who reported that they engaged in some risky sexual behaviours after circumcision. For instance, more cases compared to controls reported two or more sexual partners following circumcision (82.6% of cases compared to 68.1% of controls; *p*=0.01). Also, more cases reported inconsistent condom use than controls (67.6% vs. 36.9%, *p*<0.01) and having sex under the influence of peers (cases: 61.9% vs. controls: 28.6%, *p*<0.01). The proportion of cases that reported resuming sex before complete wound healing was 36.9% compared to 14.1% among controls (*p*<0.01). The proportion of participants who reported taking alcohol before sex was higher among cases than controls (52.7% vs. 22.4%, *p*<0.01).

Further analysis of changes in sexual behaviour following circumcision showed that after circumcision, the proportion of cases reporting sexual intercourse for the first time was higher than that of controls (81.3% of cases compared to 68.6% of controls; *p*=0.01). Also, a higher proportion of cases (69.6%) compared to controls (28.4%) reported having acquired additional sexual partners after circumcision (*p*<0.01). A bigger proportion (52.5%) of cases compared to controls (46.4%) said they did not know their HIV status at the time they had sex, although this was not statistically different (*p*=0.31). The proportion of cases that reported that they did not know the HIV status of their sexual partners was 74.8% compared to 44.3% among controls (*p*<0.01).

### Risk factors for HIV infection before circumcision

Prior to circumcision, the adjusted risk factors for HIV infection were:being in a polygamous marriage (AOR: 6.6, CI: 2.3–18.8) and belonging to the Bagisu tribe (AOR: 6.1, CI: 2.6–14.0) (Table 2).

**Table 2:** Unadjusted and adjusted risk factors for HIV infection before circumcision

Variable	Cases (HIV+), *n*=155	Controls (HIV−), *n*=155	Unadjusted OR (95% CI)	Adjusted OR (95% CI)
Age group				
18–24	35 (37.6%)	58 (62.4%)	1	1
25–29	46 (49.5%)	47 (50.5%)	1.6 (0.9–2.9)	1.7 (0.3–8.3)
30–35	74 (59.7%)	50 (40.3%)	2.5 (1.4–4.3)	2.3 (0.5–10.6)
Marital status				
Never married	61 (41.8%)	85 (58.2%)	1	1
Currently married	80 (55.2%)	65 (44.8%)	1.7 (1.2–2.7)	2.3 (0.1–77.9)
Previously married	14 (73.7%)	5 (26.3%)	3.9 (1.3–11.4)	1.6 (0.0–132.3)
Type of marriage (for currently married)				
Monogamous	47 (44.8%)	58 (55.2%)	1	1
Polygamous	33 (82.5%)	7 (17.5%)	5.8 (2.4–14.3)	6.6 (2.3–18.8)^a^
Religion				
Muslims	33 (56.9%)	25 (43.1%)	1	
Christians	122 (48.4%)	130 (51.6%)	0.7 (0.4–1.3)	
Highest education level				
Primary and below	57 (60.0%)	38 (40.0%)	1	1
Secondary education and beyond	98 (45.6%)	117 (54.4%)	0.6 (0.3–0.9)	0.9 (0.4–2.1)
Occupation				
Non-salaried earner	122 (49.2%)	126 (50.8%)	1	
Salaried earner	33 (53.2%)	29 (46.8%)	1.2 (0.7–2.1)	
Tribe				
Non-Bagisu	34 (32.7%)	70 (67.3%)	1	1
Bagisu	121 (58.7%)	85 (41.3%)	2.9 (1.8–4.8)	6.1 (2.6–14.0)^a^
Ever had sexual intercourse				
No	86 (53.8%)	74 (46.3%)	1	
Yes	69 (46.0%)	81 (54.0%)	0.7 (0.5–1.2)	
Relationship with sexual partner(s)				
Non-marital partner	60 (46.9%)	68 (53.1%)	1	
Marital partner	9 (40.9%)	13 (59.1%)	0.8 (0.3–2.0)	
Condom use during sexual intercourse				
Consistently used condom	7 (30.4%)	16 (69.6%)	1	
Inconsistently/never used condom	62 (48.8%)	65 (51.2%)	2.2 (0.8–5.7)	
Total number of sexual partners				
1	27 (41.5%)	38 (58.5%)	1	
≥2	42 (49.4%)	43 (50.6%)	1.4 (0.7–2.6)	
Consumed alcohol before sexual intercourse				
No	22 (40.0%)	33 (60.0%)	1	
Yes	21 (60.0%)	14 (40.0%)	2.3 (0.9–5.3)	
Had sexual intercourse under the influence of peers				
No	33 (39.8%)	50 (60.2%)	1	
Yes	36 (53.7%)	31 (46.3%)	1.8 (0.9–3.4)	

a Statistically significant association at multivariable analysis.

### Risk factors for HIV infection after circumcision

After circumcision, the independent risk factors for HIV infection were: being 18 years or older at circumcision (AOR: 5.0, CI: 2.4–10.2); having/resuming sexual intercourse before the wound healed (AOR: 3.4, CI: 1.6–7.3); inconsistent use of condoms during sexual intercourse (AOR: 2.7, CI: 1.5–5.1); and having sexual intercourse under the influence of peers (AOR: 2.9, CI: 1.5–5.5). Furthermore, compared to traditional/cultural circumcision, males who reported religious circumcision were less likely to be at risk of HIV infection (AOR: 0.4, 95% CI: 0.2–0.9), but there was no significant difference in the risk of HIV infection between those who were traditionally/ culturally circumcised and those who were medically circumcised (AOR: 0.4, 95% CI: 0.1–1.1) (Table 3).

**Table 3:** Unadjusted and adjusted risk factors for HIV infection after circumcision

Variable	Cases (HIV+), *n*=155	Controls (HIV−), *n*=155	Unadjusted OR (95% CI)	Adjusted OR (95% CI)
Age at circumcision				
≤17 years	25 (26.3%)	70 (73.7%)	1	1
≥18 years	130 (60.5%)	85 (39.5%)	4.3 (2.5–7.3)	5.0 (2.4–10.2)^a^
Form of circumcision				
Traditional/cultural	115 (61.2%)	73 (38.8%)	1	1
Religious	13 (27.7%)	34 (72.3%)	0.2 (0.1–0.5)	0.4 (0.2–0.9)^a^
Medical male circumcision	27 (36.0%)	48 (64.0%)	0.4 (0.2–0.6)	0.40 (0.1–1.1)
One knife used to circumcise more than one boy (for traditionally circumcised)				
No	52 (44.4%)	65 (55.6%)	1	1
Yes	67 (59.8%)	45 (40.2%)	1.9 (1.1–3.2)	0.49 (0.2–1.6)
Has ever had sexual intercourse from the time of circumcision				
No	16 (51.6%)	15 (48.4%)	1	
Yes	139 (49.8%)	140 (50.2%)	0.9 (0.4–2.0)	
Had/ resumed sexual intercourse before the wound healed				
Yes	77 (39.9%)	116 (60.1%)	1	1
No	45 (70.3%)	19 (29.7%)	3.6 (1.9–6.6)	3.37 (1.5–7.3)^a^
Relationship with sexual partner(s)				
Non-marital partner	98 (49.2%)	101 (50.8%)	1	
Marital partner	40 (50.0%)	40 (50.0%)	1.0 (0.6–1.7)	
Total sexual partners after circumcision				
1	24 (34.8%)	45 (65.2%)	1	1
≥2	114 (54.3%)	96 (45.7%)	2.2 (1.3–3.9)	1.87 (0.9–4.0)
Condom use since circumcision				
Consistently used condoms	45 (33.6%)	89 (66.4%)	1	1
Inconsistent or none	94 (64.4%)	52 (35.6%)	3.6 (2.2–5.9)	2.7 (1.5–5.1)^a^
Consumed alcohol before sexual intercourse				
No	52 (50.0%)	52 (50.0%)	1	1
Yes	58 (79.5%)	15 (20.5%)	3.9 (2.0–7.7)	1.30 (0.4–4.8)
Had sexual intercourse under the influence of peers				
No	53 (34.6%)	100 (65.4%)	1	1
Yes	86 (68.3%)	40 (31.7%)	4.1 (2.5–6.7)	2.90 (1.5–5.5)^a^

a Statistically significant association at multivariable analysis.

## Discussion

This study assessed the risk factors for HIV infection before and after circumcision among men who were circumcised through various forms of circumcision (religious, traditional and medical). Our findings show two risk factors before circumcision; being in a polygamous marriage and of the Bagisu tribe. Significant risk factors for HIV infection after circumcision were being circumcised at adulthood (18+ years), resuming sexual intercourse before wound healing, inconsistent condom use and sex under the influence of peers. Whereas the risky sexual behaviours did not vary among cases and controls before circumcision, after circumcision the cases were more likely to engage in risky behaviours, including inconsistent or no condom use, increased number of sexual partners, acquisition of new partners post-circumcision.

The increased risk of HIV infection among the Bagisu, a traditionally circumcising tribe, may be associated with increased risky sexual behaviours during circumcision seasons as reported by studies in Kanya and Uganda [9, 29]. Traditional circumcision seasons have been associated with increased risky sexual behaviours among adults and young Bagisu men including those who are not circumcised, with several young boys having their sexual debut during circumcision seasons. This scenario has also been reported among other traditionally circumcising communities [16] and underscores the need for more targeted HIV prevention messages integrated into traditional MC.

Although not statistically significant, the percentage of controls who had medical circumcision was almost double that among cases. Similarly, the percentage of those with traditional circumcision among cases was much higher than the controls. However, we found that compared to traditional circumcision, religious circumcision was associated with reduced risk of HIV infection. Our findings are consistent with another study conducted in the same region over a decade ago that found that Muslims generally had a lower HIV infection risk profile than other circumcised men [24]. Several other studies have shown a lower risk of HIV infection among Muslims [30]. The protective effect of religious circumcision could be related to various factors including the age at circumcision, religious beliefs, and practices [30–32]. Many Muslims are known to circumcise pre-puberty (infants and younger children) [31–33] which has been shown to be more protective against HIV infection than circumcising later in adulthood [31].

Traditional MC as a rite of passage into manhood is not intended for HIV prevention and certain aspects of it (such as sexual intercourse shortly after circumcision and before complete wound-healing) could undermine the potential benefits of circumcision for HIV prevention or even increase HIV risk. This study also highlights the practice of one knife being used to circumcise several men, a practice that is of serious concern in relation to transmission of HIV and other blood-borne infections [34]. Studies show that some traditionally circumcised men believe that circumcision provides complete protection [35]. This presents serious HIV prevention implications given that risk compensation post-circumcision – the tendency to engage in high risk behaviours due to a belief that circumcision offers complete protection against HIV acquisition is not addressed in traditional circumcision [16, 36]. In addition, prolonged wound healing which increases the risk of HIV infection is more prevalent among traditionally circumcised men than men circumcised through other forms of circumcision [16]. Our findings show that having sexual intercourse before wound healing and under the influence of peers were risk factors for HIV infection.

In this traditionally circumcising community, HIV prevalence is 4.1% compared to the national average of 7.3% [17]. In the same region, HIV prevalence has been found to be lower (3.6%) among circumcised than uncircumcised men (4.1%) [17], which indicates that traditional circumcision – the prevalent form of MC in the sub-region – could be protective against the risk of HIV infection (compared to uncircumcised men but is a risk when compared to other forms of circumcision). The level of protection of traditional circumcision could be further increased by integrating targeted risk reduction messages and counselling, including making HIV testing widely available to men circumcising traditionally. Strategies for SMC must consider how best to accommodate and make safer the traditional practices that exist in traditionally circumcising communities [37].

### Study limitations

Our study has some limitations. Recall of practices before and after circumcision may not be that accurate after several years. Also, this study did not comprehensively and objectively address all potential risk factors. Some of the risk factors for HIV infection might be more prevalent among certain forms of circumcision and may explain the differences in risk by type of circumcision. However, this study was not designed to address this question, and we recommend further research on traditional circumcision related risky practices. Additionally, the study did not objectively ascertain the self-reported circumcision status through a physical examination. However, the risks identified by this study are consistent with other literature and further highlight the higher magnitude of these risks among cases.

## Conclusions

Our findings show that the risk factors for HIV infection were being circumcised in adulthood and risky sexual behaviours such as resuming or having sexual intercourse before wound healing, using condoms inconsistently, and having sex under peer influence. We recommend more comprehensive risk reduction interventions that go beyond medical circumcision to be integrated into other forms of circumcision, especially the traditional circumcision.
